# Influences of pH on Gelling and Digestion–Fermentation Properties of Fish Gelatin–Polysaccharide Hydrogels

**DOI:** 10.3390/foods14152631

**Published:** 2025-07-26

**Authors:** Wanyi Sun, Qiuyu Lu, Jiajing Chen, Xinxin Fan, Shengnan Zhan, Wenge Yang, Tao Huang, Fulai Li

**Affiliations:** 1 Zhejiang–Malaysia Joint Research Laboratory for Agricultural Product Processing and Nutrition, College of Food Science and Engineering, Ningbo University, Ningbo 315211, China; 18071965076@163.com (W.S.); 18372518158@163.com (Q.L.); 18868272932@163.com (J.C.); 15888289813@163.com (X.F.); zhanshengnan@nbu.edu.cn (S.Z.); yangwenge@nbu.edu.cn (W.Y.); 2 Institute of Drug Discovery Technology, Ningbo University, Ningbo 315211, China

**Keywords:** fish gelatin, xanthan gum, κ-carrageenan, gelling properties, in vitro digestibility, intestinal microbial

## Abstract

This study systematically evaluated the effects of pH (4–10) on the gelation properties, structural characteristics, and in vitro digestion–fermentation behavior of fish gelatin (FG, 6% (*w*/*v*)) hydrogels combined with either xanthan gum (XG, 0.07% (*w*/*v*)) or κ-carrageenan (κC, 0.07% (*w*/*v*)). The results revealed that the gel strength, hardness, and chewiness of the composite gels initially increased (pH 4–6) and subsequently decreased with rising pH levels. This trend correlated with the formation of a dense gel network structure. Furthermore, as pH increased, in vitro digestibility showed a similar pH-dependent trend, with FG–XG demonstrating superior enhancement compared to FG–κC. The addition of XG and κC resulted in increased gas production and a decreased pH during fermentation. Intestinal microbiota analysis revealed that both FG–XG and FG–κC improved the abundances of *Proteobacteria* and *Bacteroidete* while reducing *Firmicutes*. Compared to FG–XG and FG, FG–κC promoted higher levels of the genera *Lachnospiraceae* and *Bacteroides*, suggesting a more favorable impact on intestinal health. These findings provide valuable insights into the pH-responsive functional properties of FG-based hydrogels and their potential applications in designing novel food matrices with enhanced nutritional and probiotic attributes.

## 1. Introduction

Gelatin, derived from the hydrolysis of collagen, is one of the most versatile and utilized gelling agents, holding significant commercial value in food industries due to its special texture and ‘melt-in-the-mouth’ sensation [1]. Currently, mammalian gelatin (MG), primarily sourced from porcine and bovine hides, as well as pork and cattle skeletons, dominates the commercial market (98.5%). However, its widespread use is hampered by concerns related to animal-derived viruses (such as prions and African swine fever) and religious considerations (both Judaism and Islam forbid the consumption of any pork-related products, while Hindus do not consume cow-related products). Fish gelatin (FG), which can be obtained from plenty of aquatic by-product processing sources, such as fish skin, scales, and bones [2], has emerged as a promising alternative [3]. Nevertheless, it is important to note that the gelling and rheological characteristics of FG lag behind those of MG [4], primarily due to its low proline and hydroxyproline contents. This limitation significantly restricts its commercial applicability. To address these challenges, various scientific endeavors have been undertaken, encompassing chemical modifications such as phosphorylation, aldehyde, and phenol modifications, as well as physical modifications involving the addition of electrolytes or non-electrolytes and mechanical treatments [5]. Among these strategies, research has highlighted the efficacy of electrostatic and hydrogen bond interactions between anio-polysaccharide and gelatin molecules, which have shown particular promise in enhancing the gelling and rheological properties of FG [6].

Commonly, κ-carrageenan (κC), konjac glucomannan (KGM), Arabic gum (AG), and xanthan gum (XG) are typically selected to regulate the gelling properties of proteins. Different types of polysaccharides usually feature different structural characteristics, which further affect their application value. One of them, κC, is a hydrophilic colloid with a chemical structure composed of galactose and dehydrated galactose polysaccharide sulfate calcium, potassium, sodium, and ammonium salt [7]. Our previous studies have highlighted κC’s ability to enhance the gelling properties of FG hydrogels, including gel strength, hardness, and viscosity [8]. Xanthan gum (XG) is a microbial exopolysaccharide produced by Xanthomonas canola through fermentation engineering and is valued for its excellent water solubility and compatibility with other substances [9]. In the food industry, XG functions as a thickening agent, suspension agent, emulsifier, and stabilizer. Research findings indicate that XG effectively improves the hardness of gelatin gels [10]. Both κC and XG, which are negatively charged polysaccharides, enhance the gelling properties of FG through electrostatic interactions. This enhancement is significantly influenced by factors such as pH, salt concentration, and the ratio of FG to polysaccharide [11]. Notably, there are limited comparative studies exploring the mechanisms by which pH and different types of polysaccharides affect these interactions, highlighting an area that warrants further investigation. Furthermore, electrostatic interactions have been recognized not only for their ability to enhance protein gelation, but also for their impact on protein digestibility [12]. However, existing research is deficient in comparatively investigating how the two negatively charged polysaccharides, κC and XG, influence the in vitro digestibility of FG and the characteristics of intestinal microbiota.

Therefore, the primary objective of this study is to conduct a comparative analysis of the effects of κC and XG on the gel and structural properties of FG under varying pH conditions. Additionally, we aim to examine the in vitro digestion characteristics of the prepared gelatin gels and their effect on human intestinal microbiota through in vitro fermentation experiments. By comprehensively assessing the physicochemical and physiological properties of FG influenced by these two polysaccharides, we anticipate that our findings will provide valuable insights for the food industry in making informed choices regarding the use of additives in the future.

## 2. Materials and Methods

### 2.1. Materials

Fish gelatin (FG, containing 88.14 ± 1.57% crude protein, 4.78 ± 0.03% ash, and 7.93 ± 0.63% moisture, *w*/*v*) and κ-carrageenan (κC, with a molecular weight of 20–0 × 10^4^ and viscosity in H_2_O of approximately 52 cP) were procured from Shanghai Yuanye Biotechnology Co., Ltd (Shanghai, China). Xanthan gum (XG, purity 85%, viscosity in 1% KCl ≥ 1200 cP) was obtained from Solarbio Science & Technology Co., Ltd. (Beijing, China). O-Phthalaldehyde (purity ≥ 99%) reagent was obtained from Shanghai Aladdin Biochemical Technology Co. (Shanghai, China) Pepsin (>3000 U/mg) was purchased from Shanghai Macklin Biochemical Co., Ltd. (Shanghai, China). All other chemicals used were of analytical grade, and deionized water was utilized for all experiments.

### 2.2. Preparation of Gelatin Solution

A 6% (*w*/*v*) FG solution was prepared by dissolving FG powder in distilled water by heating using a water bath at 40 °C for 2 h. Subsequently, 0.07% (*w*/*v*) of XG and κC, respectively, were added separately to the FG solution, resulting in the formulation of FG–XG and FG–κC solutions. The mixture was stirred at 100 rpm until complete dissolution. The pH of the mixture was adjusted to 4, 5, 6, 7, 8, and 9 using 1.0 M NaOH and HCl solutions at room temperature and verified using a pH meter. The prepared samples were named according to the pH value and stored at 4 °C for subsequent experiments.

### 2.3. Gelling Properties Analysis

#### 2.3.1. Determination of Gel Strength

The gel strength of all hydrogels was determined using the method described in [13], employing Texture Profile Analysis (TPA) (Stable Micro system, Surrey, UK) equipped with a P/0.5 probe. A 15 mL gelatin solution (6%, *w*/*v*) was incubated at 10 °C for 16–18 h. Gel strength was quantified as the maximum force exerted when the probe penetrated the colloid to a depth of 4 mm at a speed of 1 mm/s. Each sample underwent three parallel measurements to ensure accuracy and reliability.

#### 2.3.2. Determination of Textural Properties

The textural properties of samples were assessed using TPA (Stable Micro system, Surrey, UK) utilizing a P/50 probe, following the method outlined by Min et al. [14]. All samples (6%, *w*/*v*) were incubated in a refrigerator at 10 °C for 16–18 h. The resulting gels were then shaped into cylinders with a height of 1.5 cm and a diameter of 2.5 cm. The test parameters included a compression of 40%, an induction force of 5 g, and a test speed of 1 mm/s. Each sample underwent three parallel measurements to ensure accuracy and consistency.

### 2.4. Rheological Behavior Analysis

#### 2.4.1. Determination of Apparent Viscosity

Shear rate tests of the gelatin solution (6%, *w*/*v*) were conducted using a DHR-2 rheometer (Discovery Hybrid Rheometer, TA Instrument, Newcastle, Delaware, USA) with a stainless-steel parallel plate (diameter: 40 mm, gap: 1 mm). The tests were performed at a fixed temperature of 25 °C, with shear rates ranging from 0.01 to 1000 s^−1^ [8]. The flow behavior of each sample was evaluated using the Power law model, τ = kγ^n^, where τ represents the apparent viscosity (Pa·s), γ denotes the shear rate (s^−1^), k is the consistency index, and n is the flow behavior index.

#### 2.4.2. Frequency Sweep

All gelatin solutions underwent maturation at 5 °C for 1 h. Subsequently, the storage modulus (G′) and loss modulus (G″) were measured across a frequency range of 0.01–10 Hz, employing a 1% strain. The entire process was executed within the determined linear viscoelastic region [15].

### 2.5. Structural Properties Analysis

#### 2.5.1. Fourier Infrared Spectroscopy (FTIR)

The group absorption peak characteristics of the samples were examined using Fourier Transform Infrared Spectroscopy (PerkinElmer, Waltham, MA, USA). Lyophilized gelatin (4 mg) was thoroughly mixed with KBr powder (120 mg) and manually pressed into tablets using a tablet press. The spectra were subsequently scanned over the range from 4000 to 400 cm^−1^ with a resolution of 4 cm^−1^.

#### 2.5.2. Environmental Scanning Electron Microscopy

The freeze-dried samples were affixed to a sample stage using conductive tape and subsequently examined for their gel network structure utilizing an environmental scanning electron microscope (ESEM) (S-3400 N, Hitachi Ltd., Tokyo, Japan) in low-vacuum mode. The testing parameters included a voltage of 150 kV and a magnification set as ×320, as per the approach outlined by Geng et al. [16].

### 2.6. Determination of In Vitro Digestive Characteristics

#### 2.6.1. Simulated Stomach Digestion

In vitro digestion was performed according to the methods described by Wang et al. [15]. A gelatin hydrogel sample (2 g) was added to 8 mL of phosphate buffer (PBS, 10 mM, Na_2_HPO_4_-NaH_2_PO_4_, pH 7.0) and homogenized at 8000 r/min to create a homogeneous mixture. The pH of the mixture was adjusted to 2.0 using 1 M HCl, and pepsin (2500 U/mg) was added at a ratio of 1:50 (*w*/*w*). Simulated in vitro gastric digestion was conducted at 37 °C with a rotational speed of 50 rpm. During gastric digestion, 2 mL samples were collected at 60 min and 120 min. Subsequently, the pH of the solution was neutralized to 7.5 using 1 M NaOH. Then, trypsin (250 U/mg) was introduced to each sample at a ratio of 1:50 (*w*/*w*), and the mixture was subjected to intestinal digestion conditions at 50 rpm. During intestinal digestion, 2 mL samples were collected at both 60 min and 120 min. The obtained mixture was heated by boiling water for 10 min to inactive the enzyme.

#### 2.6.2. Determination of Free Amino Acid (FAA) Content

The FAA content of the digestion solution was quantified to assess the peptide concentration, following the procedure described by Moretton et al. [17]. Specifically, 50 μL of digestive fluid was mixed with 1 mL of OPA reagent and allowed to react for 2 min. Subsequently, the absorbance was measured at 340 nm using a UV–visible spectrophotometer. Leucine was utilized to construct the standard curve, and each sample was subjected to three parallel measurements to ensure accuracy.

### 2.7. Determination of Fermentation Characteristics

#### 2.7.1. In Vitro Fermentation

Five healthy, non-vegetarian volunteers without intestinal diseases were recruited for the study. Fresh feces from these volunteers were collected and mixed in a beaker, then diluted to 15% with PBS. This mixture was filtered through four layers of sterile gauze folded in a beaker to obtain a feces slurry. The supernatant of the digestive liquid supernatant (1 mL) was pre-added to a 2 mL penicillin bottle. In the control group, distilled water was used to replace the sample, and 8 mL of feces slurry was added to the penicillin bottle along with the existing samples. The penicillin bottle was sealed with both a sealing cap and an aluminum cap to ensure airtightness. The bottles were incubated in a water bath at 37 °C with manual shaking. Fermentation samples were collected at 0, 6, 12, 18, and 24 h, and the reaction was stopped in an ice bath. The fermentation solution was stored at −80 °C for subsequent analysis [18].

#### 2.7.2. Determination of Gas Generation During In Vitro Fermentation

The gas production of each sample during fermentation was measured using disposable syringes. The specific procedure involved swiftly inserting the syringe into the rubber cap of the penicillin bottle, and the height of the piston rise was recorded as the gas production (mL).

#### 2.7.3. Determination of pH During In Vitro Fermentation

After in vitro fermentation, the samples were placed in an ice bath for 20 min and centrifuged at 10,000 rpm for 10 min. The pH value of each sample’s supernatant was measured using a benchtop pH meter S400 (Mettler-Toledo, LLC, Columbus, OH, USA). The measurements were repeated three times.

#### 2.7.4. Microbial Diversity Analysis

The genomic DNA of the fermentation precipitates was extracted using a Genomic DNA Extraction Kit. The bacterial 16s rDNA V3–V4 region and the fungal ITS1–ITS2 region gene sequences were amplified using PCR. Following amplification, the integrity of the PCR products was assessed using 1.8% agarose gel electrophoresis. Subsequently, the library concentration was measured, and sequencing was performed using the Illumina NovaSeq 6000 platform. The high-throughput sequencing data were analyzed using Trimmomatic-v0.33 software and Cutadapt 1.9.1 software. Functional predictive analysis was conducted using PICRUSt2, which predicts the functional richness of samples based on the abundance of annotated gene sequences within them.

### 2.8. Statistical Analysis

Each experiment was replicated more than three times, and the results are presented as mean ± standard deviation. Statistical significance was assessed using one-way analysis of variance (ANOVA) and Tukey multiple comparisons with SPSS 21.0 software, with *p* < 0.05 considered statistically significant. Data visualization was carried out using Origin 2018 (trial edition). In the statistical analysis, different letters were used to denote significant differences, with significance established at *p* < 0.05.

## 3. Results and Discussion

### 3.1. Gel Strength and Textural Properties

Gel strength is a crucial parameter that reflects the quality of a gel, indicating its stability and strength, which is particularly significant in practical food applications [16]. According to our previous report [2,4], FG possesses a negative charge. Theoretically, both negatively charged FG and anio-polysaccharide (AP) should repel each other due to electrostatic repulsion, thus preventing the formation of complexes and resulting in lower gelling properties. As shown in Table 1, compared to the original sample, the gel strength, hardness, gumminess, and chewiness of the FG–XG and FG–κC systems significantly improved. The gelling properties of both systems initially increased and then decreased with a rising pH. This suggests that the two polymers could still interact to form stable FG–AP complexes due to electrostatic interactions, thereby enhancing the gelling properties of the two FG–AP systems. Herein, the isoelectric point (IP) value of FG was approximately 5.40. In the FG–κC system, the highest gelling properties were observed at pH 6, while in the FG–XG system, they peaked at pH 5. The pH impacted gelling properties by altering the net charge of the protein [19]. At pH levels below the IP of the protein, proteins and AP exhibit opposite net charges, leading to maximum electrostatic attraction [20]. Consequently, FG–κC6 and FG–XG5 exhibited the highest electrostatic repulsion force or electrostatic reaction force, promoting the formation of stable gel network structures. However, as pH was further increased, excessive OH^−^ ions and AP repelled each other, causing the FG molecules and AP to separate, consequently reducing gel strength. Moreover, the elevation of pH weakened the formation of hydrogen bonds, further decreasing gel strength [21]. Interestingly, within the pH range of 6.0–9.0, there was a non-significant effect on the gel strength of FG–XG. This difference may be attributed to the structural heterogeneity of κC and XG. Additionally, at a low pH (4.0), FG–XG exhibited a higher gel strength than FG–κC, which can be attributed to the loss of gel strength in κC due to self-hydrolysis. Conversely, at a high pH (6.0–9.0), FG–κC demonstrated a higher gel strength than FG–XG, as both XG and κC are negatively charged, resulting in electrostatic repulsion with FG. Furthermore, the higher charge carried by XG led to greater electrostatic repulsion between XG and FG, limiting molecular polymerization and resulting in a lower gel strength [22].

Texture profile analysis (TPA), which involves compressing a sample twice, is crucial for evaluating the texture and quality of a colloid [23]. TPA is primarily influenced by amino acid composition, molecular weight distribution, and molecular aggregation. The hardness values of the FG–XG and FG–κC systems peaked at pH 5 and 6, respectively, which corresponded with the observed gel strength results. Under low-pH conditions, the positively charged surfaces of the FG and AP molecules exhibited enhanced attraction to negatively charged groups, significantly improving their cross-linking capacity and thereby enhancing gel toughness. Conversely, under high-pH conditions, the negative charge on FG molecules induced repulsive forces against positively charged components, leading to a reduction in cross-links and a decrease in structural strength, resulting in brittle and hard characteristics. The trends in hardness, gumminess, and chewiness, which initially increased and then decreased with pH, aligned with the gel strength and pH trends when reaching the peak. Moreover, both FG–XG and FG–κC exhibited higher springiness values than that of FG, while pH had a non-significant influence on the resilience, springiness, and cohesiveness values of the FG–AP systems. The index of springiness is a measure of how much the gel structure is broken down by the initial compression. Higher springiness requires more mastication energy in the mouth. Therefore, the addition of AP can alter the textural properties of FG, especially for the alteration of pH.

### 3.2. Analysis of Rheological Behaviors

#### 3.2.1. Apparent Viscosity

**Figure 1:**
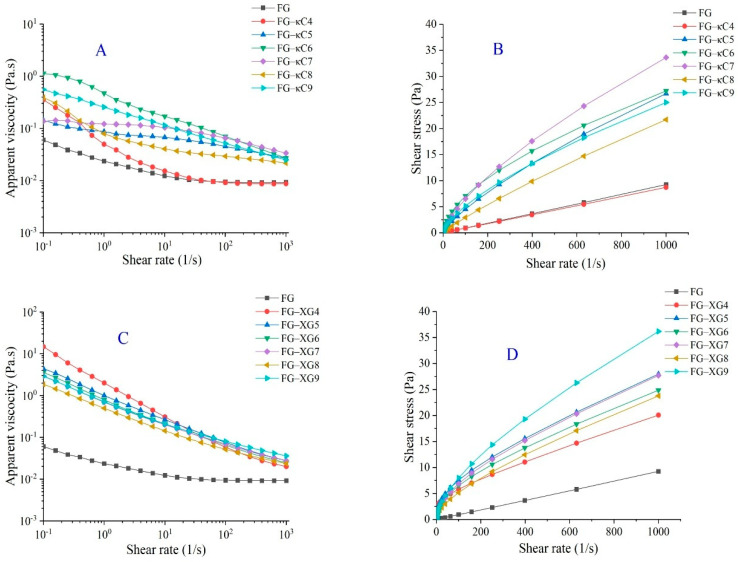
The apparent viscosity and shear stress of FG–AP complex solutions under varying pH conditions. (**A**) Apparent viscosity of FG–κC under pH conditions ranging from 4 to 9. (**B**) Shear stress of FG–κC complex solutions under pH conditions ranging from 4 to 9. (**C**) Apparent viscosity of FG–XG under pH conditions ranging from 4 to 9. (**D**) Shear stress of FG–XG complex solutions under pH conditions ranging from 4 to 9.

Viscosity is a crucial parameter in the food processing industry, affecting processing, taste, quality, and shelf life. pH exerts a significant impact on apparent viscosity [24]. As shown in Figure 1A,C, both XG and κC enhanced the apparent viscosity of FG. This enhancement could be attributed to the addition of AP, which increased the total substance in the solution and facilitated the formation of numerous aggregates through the formation of hydrogen bonding and electrostatic interactions, leading to a shear dilution phenomenon [25]. FG–AP complexes exhibited a significantly higher viscosity, with FG–XG5 and FG–κC6 displaying the highest values within their respective formulation systems. This increase could be attributed to the synergistic effects of larger inter-molecular electrostatic forces and stronger hydrogen bonding, which collectively enhanced the resistance to flow in the gelatin solution, resulting in a high viscosity. The trend of shear stress with shear rate reflected that of viscosity; at low shear rates, the system experienced less shear stress (Figure 1B,D), maintaining a stable liquid state and resulting in a higher viscosity. Conversely, higher shear rates disrupted the hydrogen bonds within the FG–AP gel network, causing the disintegration of the network structure and a decrease in apparent viscosity [26].

The η_50_ value is associated with the consistency and lubricity of food products. Generally speaking, the viscosity (η_50_ value and a value) of FG–κC and FG–XG initially increased and then decreased, with FG–κC6 and FG–XG5 exhibiting the highest values within their respective systems. This trend might be have been due to the fact the electrostatic and steric repulsion increased firstly and then decreased, changing the resistance ability of the systems to flow (hence, a higher viscosity) [27]. The η_50_ initially increased and then decreased with an increasing pH, consistent with the trends observed in the texture and gel properties. In addition, FG–κC demonstrated a higher viscosity than FG–XG at the same pH, with FG–κC6 displaying the highest η_50_ value. This suggests that the addition of κC significantly facilitated interaction with FG via inter-molecular attraction, thereby enhancing the thickening and stabilizing properties of FG [28]. Furthermore, the apparent viscosity results were fitted using the Power law model (Table S1), revealing R^2^ values exceeding 0.95, which indicates the suitability of the model for data fitting in this sample. Additionally, the results (Table S1) showed that the k values in the FG–κC system ranged from 0.009 to 0.461 Pa.s^n^. The k values initially increased with a rising pH and then decreased. Conversely, the flow behavior index (n) first decreased and then increased with pH ranges from four to nine. In the FG–XG system, the k values initially decreased from 0.946 to 0.258 Pa.s^n^ and then increased as pH increased, while n increased from 0.427 to 0.647 within the same pH range. Regardless of whether they were in the FG–κC or FG–XG system, the n values of all samples were <1.0, indicating that all samples exhibited pseudoplastic behavior. The variations in the k and n indexes may have resulted from the structural changes in FG–AP under different pH conditions.

#### 3.2.2. Frequency Sweep

**Figure 2:**
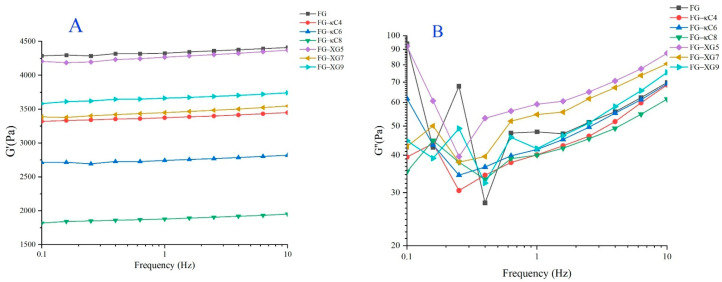
Frequency sweep of FG–AP complex gels. (**A**) Frequency sweep of FG–κC under pH conditions ranging from 4 to 9. (**B**) Frequency sweep of FG–XG under pH conditions ranging from 4 to 9.

Frequency tests are essential for characterizing the gel strength of composite colloids, facilitating the evaluation of product quality during application processes [29]. G′ is closely associated with the formation of triple helix content. As shown in Figure 2A, G′ increased with frequency, displaying a pronounced frequency dependence. Conversely, G″ decreased first and then increased with frequency (Figure 2B). Additionally, G′ exhibited a decreasing trend with an increasing pH, with the G′ of FG–XG significantly surpassing that of FG–κC (Figure 2A). This suggests that FG–XG demonstrated superior elasticity compared to FG–κC. However, the G′ of all FG–AP complexes was lower than that of the original samples (Figure 2A). This could be attributed to the fact that the addition of XG and κC, which improved the viscosity of the solution, reduced the self-aggregation of FG and ultimately inhibited the formation of a triple helix structure, leading to a decrease in G′ [15].

Furthermore, the frequency scan results were fitted using the Power law model (Table S2), revealing R^2^ values exceeding 0.90, which indicates the suitability of the model for data fitting. The G_0_ value of FG–XG surpassed that of FG–κC, signifying a stronger colloidal strength in FG–XG, consistent with the gel strength and TPA results. The overall G′/G value of FG–XG was larger, indicating a stronger gel network structure compared to FG–κC.

### 3.3. Structural Analysis

#### 3.3.1. FTIR Analysis

**Figure 3:**
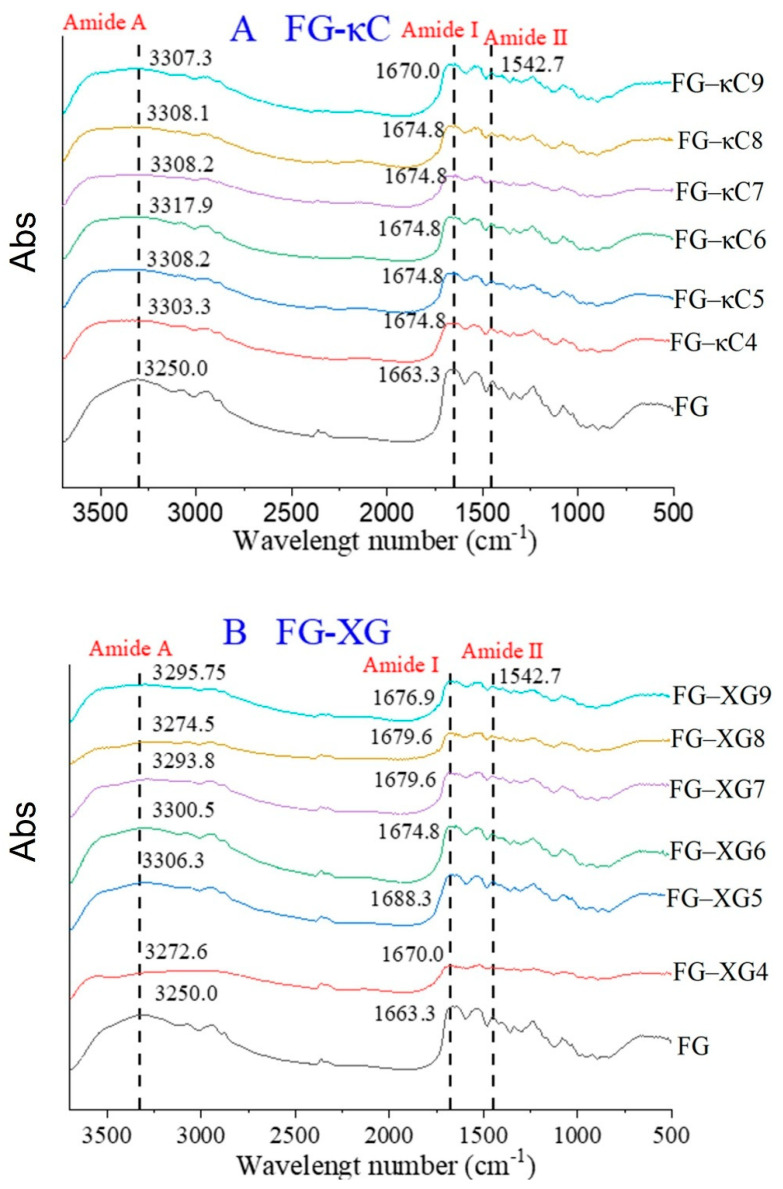
FTIR spectra of FG–AP complex under varying pH conditions. (**A**) FTIR spectra of FG–κC under pH conditions ranging from 4 to 9. (**B**) FTIR spectra of FG–XG under pH conditions ranging from 4 to 9.

FTIR is a widely utilized technique for protein structural analysis. Gelatin molecules typically exhibit characteristic absorption peaks and areas around 3250–3450 cm^−1^ (Amide A), 1600–1700 cm^−1^ (Amide I), and 1500–1550 cm^−1^ (Amide II) [30]. As shown in Figure 3A,B, all samples exhibited a similar FTIR spectrum, indicating that pH, XG and κC had no remarkable impact on the functional groups of gelatin. For the FG–κC (Figure 3A) and FG–XG (Figure 3B) systems, all gelatin samples showed higher Amide A values compared to FG. Moreover, the Amide A values increased first and then decreased with an increase in pH, with FG–κC6 and FG–XG5 exhibiting the highest values in their respective groups. The higher Amide A values suggest a great number of inter-molecular hydrogen bonds formed between the O-H groups in the AP and the N-H groups of FG [18].

The peak of Amide I represents C=O stretching or hydrogen bond coupling with COO and is related to protein conformation and hydrogen bond strength [31]. The position of the Amide I peaks of the FG–κC system remained relatively consistent across different pH levels (Figure 3A), indicating a similar hydrogen bond content. However, the Amide I peak of FG–XG initially increased and then decreased with pH elevation (Figure 3B), and FG–XG5 exhibited the highest peak values, suggesting the greatest hydrogen bond content, consistent with its gelling properties (Table 1). This indicates that hydrogen ion concentration affected the electrostatic interaction between FG protein’s hydrogen bond and XG’s carboxyl group. Additionally, data from the Amide I region clearly indicated significant differences between low- and high-frequency β-sheet structures, random structures and α-helix, and β-turn structures (Table S3). Amide II results from N-H bending vibrations and C-N tensile vibrations [13]. The wave number of Amide II in all samples remained consistent at 1542.7 cm^−1^ (Figure 3A,B), indicating that the addition of the two APs had no effect on FG’s Amide II band.

#### 3.3.2. Gels Network

**Figure 4:**
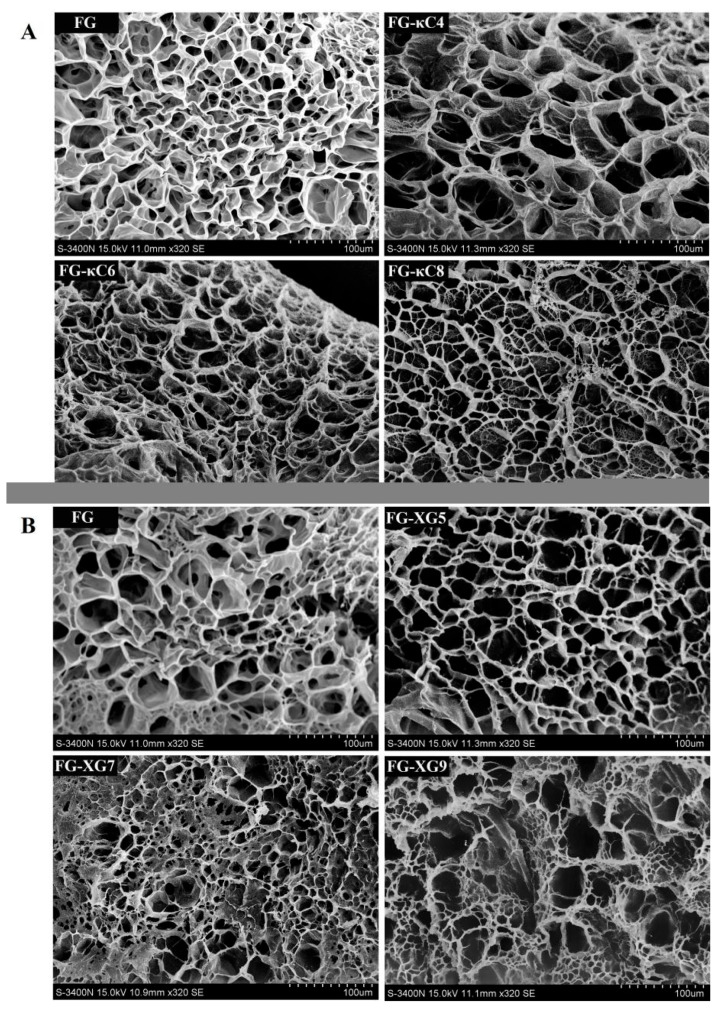
Characterization of FG–AP gel microstructures using scanning electron microscopy (SEM). (**A**) Microstructure of FG–κC gels. (**B**) Microstructure of FG–XG gels.

The micro-structure of the composite colloid was analyzed using environmental scanning electron microscopy. As per the results depicted in Figure 4, the micrographs revealed that FG alone was significantly different from FG–AP, and the microstructure of FG–AP varied under different pH conditions. This indicates that both the addition of anionic polyanions and the pH level affected the structure of FG. In the FG–κC system (Figure 4A), the network structure of the composite colloid appeared denser at pH 6.0 compared to pH 4.0, and the pore size of FG–κC6 was more uniform than that of FG–κC8 (Figure S1). In contrast, in the FG–XG system (Figure 4B), the network structure of the composite colloid exhibited better organization and stability at pH 5.0, consistent with the results of the gel strength measurements. Gelatin molecules entrapped polysaccharide molecules within a three-dimensional mesh structure, leading to an ordered gel structure. This ordered arrangement of gel molecules and their aggregation significantly contributed to the improvement of the gel strength of the colloids. However, when the pH increased to 9.0, the structure of the FG–XG9 network was visibly destroyed, showing larger and unevenly distributed cavities or pores (Figure 4B and Figure S1). Additionally, the average pore sizes values of FG, FG–κC4, FG–κC6, and FG–κC8 were 38.34 ± 7.81, 31.75 ± 2.62, 19.91 ± 5.98, and 22.12 ± 6.70 μm, respectively. The average pore size values of FG–XG5, FG–XG7, and FG–XG9 were 20.16 ± 6.34, 25.48 ± 9.62, and 23.70 ± 5.27 μm, respectively. A smaller pore size correlated with higher gelling properties. Thus, the appropriate addition of κC and XG resulted in a denser gel network of gelatin molecules, effectively enhancing the gelling properties of gelatin [32].

### 3.4. Analysis of Digestive Properties

**Figure 5:**
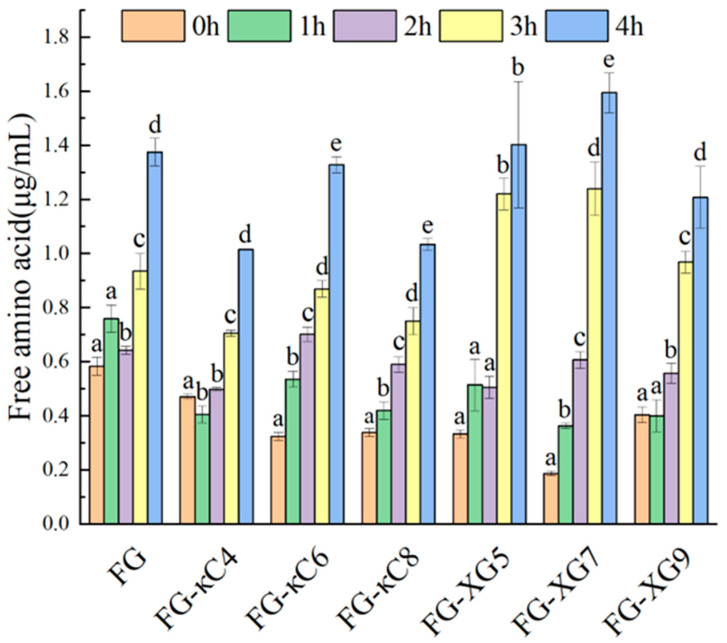
The content of free amino acids in FG–AP complex at different pH values. Different lowercase letters indicated significant difference between groups (*p* < 0.05).

The OPA method is employed to quantify the primary FAAs in gastric and intestinal digestive fluids, reflecting the extent of protein digestion. Figure 5 illustrates that the FAA content of the samples generally increased with digestion time. Normally, during the gastric digestion stage, FG showed the highest FAAs compared to FG–κC and FG–XG. During gastric digestion (acid condition), κC and XG interacted with FG to form stable complexes that buried interaction and enzymolysis sites, thereby hindering their breakdown [33]. Furthermore, FG–κC exhibited a superior digestive efficiency compared to FG–XG, especially FG–κC6. This suggests that FG–κC was capable of forming more absorbable proteins and bioactive peptides than FG–XG [34]. This effect could potentially be attributed to the addition of XG, which altered the structural properties of FG, exposing more enzyme hydrolysis sites. FG is a macromolecular substance composed of various amino acids with lots of sites that can interact with stomach acid and pepsin. Additionally, κC contains more α-L-galactoic acid, an oligosaccharide possibly promoting digestion in the human body. Conversely, XG lacks such oligosaccharides.

During intestinal digestion, the FAA contents of all samples increased with digestion time. The FG–κC group showed lower FAA levels compared to FG, while FG–κC6 presented the highest FAAs within its group. Conversely, FG–XG demonstrated significantly higher FAAs than those of FG, with FG–XG7 exhibiting the highest FAA values. This might have been due to the fact that the alkalinity condition may easily loosen the structure of FG–XG, contributing to an increased digestion ability. Overall, compared with FG–XG, FG–κC showed a lower digestibility, which is also associated with a denser gel network (Table 1).

### 3.5. Analysis of Fermentation Characterization

#### 3.5.1. Gas Production During the Fermentation Process

**Figure 6:**
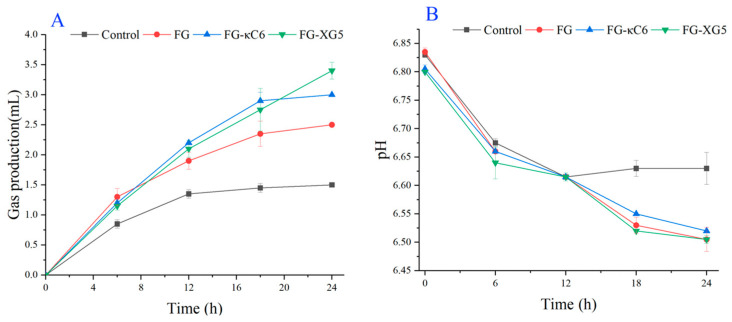
Gas production and pH changes during fermentation of FG, FG–κC6, and FG–XG5. (**A**) Gas production during fermentation process. (**B**) pH values at different fermentation times.

After proteins and other substances are fermented by intestinal microbiota, the gases produced primarily consist of carbon dioxide, methane, hydrogen, and hydrogen sulfide [35]. The changes in gas production during fermentation following the colloidal digestion of FG, FG–κC, and FG–XG are illustrated in Figure 6A. During the initial 0–6 h of fermentation, gas production increased rapidly, indicating vigorous metabolic activity of the bacterial colony. In the middle stage of fermentation (6–12 h), gas production continued to increase but at a slower rate. After 24 h of fermentation, both the FG–κC and FG–XG groups exhibited higher gas production compared to the FG and control groups, with the FG–XG group showing the highest gas production. This suggests that the addition of XG and κC could promote the colon fermentation of FG, with XG being more effective in this regard.

#### 3.5.2. pH Changes During Fermentation

The fermentation and breakdown of proteins by intestinal microorganisms result in the production of acids, such as lactic acid and short-chain fatty acids, leading to a decrease in pH in the colonic region, thereby affecting the intestinal microbial community in the large intestine [36]. As shown in Figure 6B, the pH of the fermentation broth was 6.8, indicating a weakly acidic environment. Before the start of fermentation, there was no significant difference in the pH values between the experimental group and the blank group. As fermentation progressed, the pH of all groups showed a decreasing trend. However, after 12 h, the pH continued to decline in the experimental and control groups, while the pH of the blank group remained relatively stable.

The decrease in the pH of the fermentation broth was attributed to the utilization of carbohydrates by microorganisms during fermentation, which led to the formation of short-chain fatty acids and other acidic substances, thereby reducing the pH of the entire system. After 24 h of fermentation, the pH of the experimental and control groups was lower than that of the blank group. Therefore, based on the pH changes observed in the fermentation system, it can be hypothesized that FG, FG–XG, and FG–κC could lower the pH of the fermentation system, thereby influencing the microbial community composition and the metabolic activities in the fermentation broth. Interestingly, after 12 of fermentation, FG–XG5 showed a lower pH than that of FG–κC6. XG functions as insoluble dietary fiber, and it can be adsorbed by beneficial bacteria like bifidobacteria and contains hydroxyproline, which could hydrolyze protein to form biologically active amino acids, further lowering pH.

### 3.6. Microbial Diversity Change

#### 3.6.1. Alpha Diversity Analysis

Alpha diversity analysis allows for the assessment of microbiota diversity based on species abundance and evenness. Indices such as Chao 1 and ACE exhibited positive correlations with community richness, while the Shannon and Simpson indices reflected both community diversity and evenness across various samples. As depicted in Figure S2, the Chao 1 and ACE indices of the FG group were higher compared to those of the FG–κC and FG–XG groups. This suggests that the addition of κC and XG to FG diminished the abundance of bacteria in the human intestinal tract. Furthermore, the Shannon and Simpson indices, which reflect microbiota diversity, demonstrated minimal variation among the different groups [37].

#### 3.6.2. Intestinal Microbial Community Analysis

**Figure 7:**
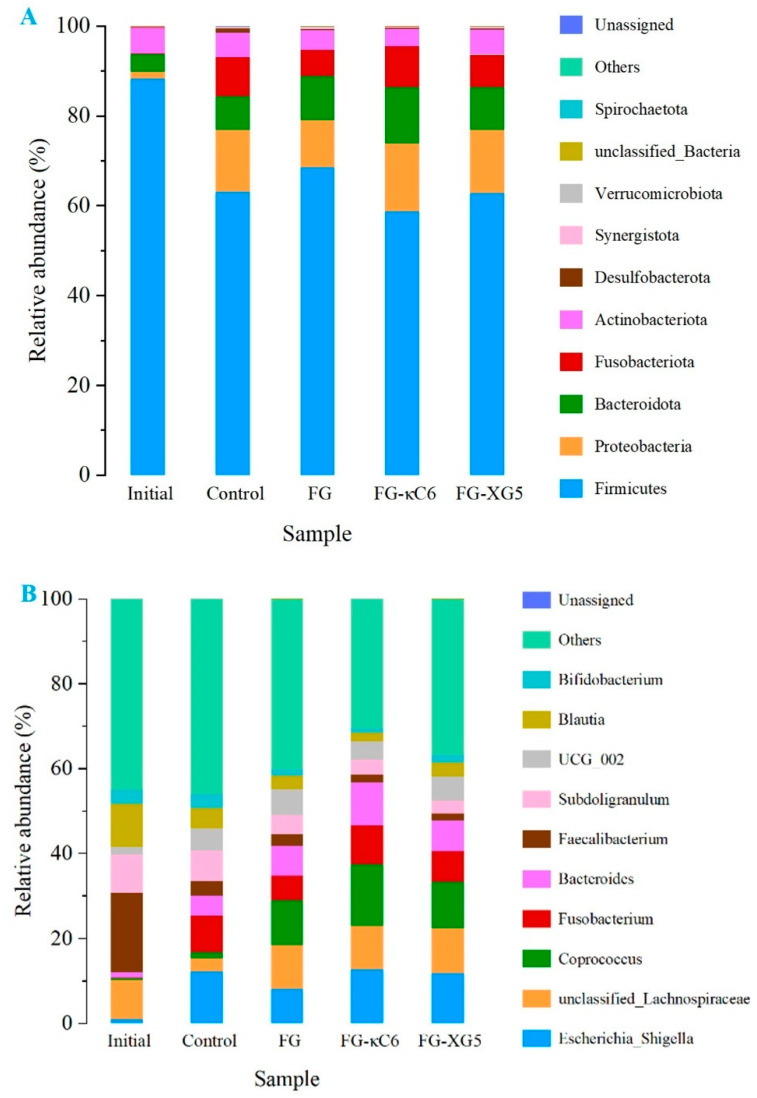
Gut microbial composition at phylum level (**A**) and genus level (**B**) in the fermentation fluids of different samples after fermentation.

The microbial composition at both the phylum and genus levels in the fermentation solutions of different samples after fermentation was identified. Principal component analysis and hierarchical cluster analysis demonstrated that each group could be distinctly clustered and well differentiated (Figure S3A). As Figure 7 illustrates, each color represents a distinct species, with the top ten species by abundance displayed in the figure, while others are combined into the “Others” category. At the phylum level (Figure 7A and Figure S4A), the intestinal microbiota was predominantly composed of *Actinobacteria*, *Proteobacteria*, *Bacteroidetes*, and *Firmicutes*, accounting for over 98.55% of the microbial population. Among these, *Firmicutes* and *Bacteroidetes* are key producers of short-chain fatty acids, primarily through the breakdown of complex carbohydrates. Notably, *Bacteroides* is a major producer of propionic acid in the colon, which plays a role in regulating blood lipids and cholesterol levels [37]. *Firmicutes* was the main phyla in blank group (Initial), accounting for 88.29%. Compared to the blank sample (Initial), after 24 h of fermentation, the relative content of *Bacteroidetes* and *Proteobacteria* in the fermentation solution of all samples increased, while the content of *Firmicutes* and *Actinobacteria* decreased. Compared with FG group, FG–XG5 had a lower *Firmicutes* content (62.83%), while FG–κC6 had lower *Firmicutes* (58.80%) and *Actinobacteria* contents (3.77%). Conversely, FG–XG5 had higher contents of *Proteobacteria* (13.98%) and *Bacteroidetes* (9.64%) compared to the FG (10.41%) and control (7.60%) groups, respectively. FG–κC6 had higher contents of *Proteobacteria* (15.10%) and *Bacteroidetes* (12.53%) compared to the FG and control groups. *Firmicutes* and *Bacteroidetes* have been associated with certain diseases such as obesity and diabetes, with obese individuals typically exhibiting higher levels of *Firmicutes* and lower levels of *Bacteroidetes* compared to healthy individuals [38]. Thus, the digestive products of FG–κC6 and FG–XG5 can help to regulate intestinal microorganisms and promote intestinal health.

At the genus level (Figure 7B and Figure S4B), the abundances of *Faecalibacterium* and *Blautia* (initial) significantly decreased after 24 h of fermentation. In contrast, *UGG 002*, *Escherichia Shigella*, *Bacteroides*, *Fusobacterium*, and *Coprococcus* showed significant increases. Compared to the control group, the FG, FG–κC6, and FG–XG5 groups exhibited higher relative abundances of *Bactcroides*, *Lachnospiraceae*, and *Coprococcus*, but lower levels of *Bifidobacterium*, *Faecalibacterium*, and *Subdoligranulum*. Furthermore, the abundances of genera in the FG, FG–κC6, and FG–XG5 groups also differed. Notably, FG–κC6 demonstrated the highest levels of *Coprococcus* (14.53%) and *Bactcroides* (10.09%) and higher levels of *Lachnospiraceae* (10.19%). *Bactcroides* and *Lachnospiraceae* are potentially beneficial bacteria involved in the metabolism of a variety of carbohydrates, particularly pectin (a complex dietary fiber and prebiotic) found in fruits and vegetables [39]. Their fermentation leads to the production of acetic acid and butyric acid, which serve as the primary sources of energy for the host. Additionally, *Coprococcus* is recognized as a key microorganism in the production of butyric acid [40]. Therefore, the increase in the abundances of *Bactcroides*, *Lachnospiraceae*, and *Coprococcus* may be a primary factor contributing to the decrease in the pH of the fermentation broth. In summary, both FG–κC6 and FG–XG5 could increase the abundance of beneficial bacteria, demonstrating high application potential for products such as yoghurt and surimi gels.

#### 3.6.3. Functional Predictive Analysis

**Figure 8:**
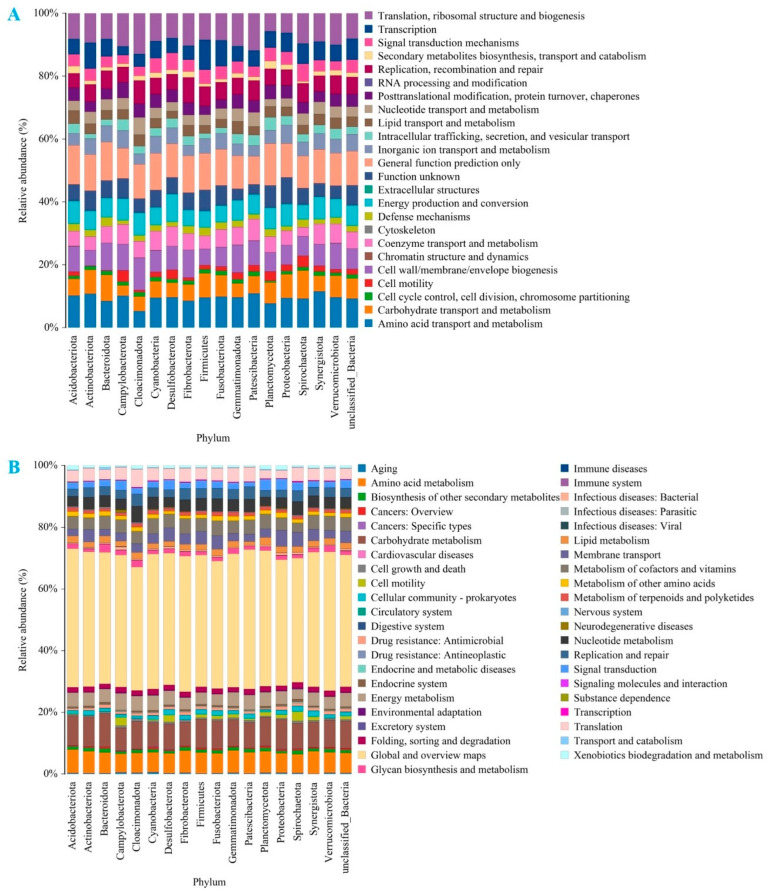
Functional predictive analysis of microbial genes present in fermentation liquids. (**A**) COG functional classification of intestinal microbiota in fermentation fluids. (**B**) KEGG functional classification of intestinal microbiota in fermentation fluids.

The quality of food is closely linked to the metabolic pathways of microorganisms. Predicting metabolic pathways can be achieved by analyzing the functional genes of microorganisms present in fermentation liquids [41]. Gene information from unknown species can be inferred based on the gene type and abundance information of known species. By integrating this information with COG pathway data, the pathways of the entire microbial community can then be predicted. Through the analysis of COG metabolic pathways, the differences and changes in the functional genes of microbial communities can be observed across different samples. This approach provides an effective means to study how microbial communities adapt their metabolic functions to environmental changes [42]. Figure 8A and Figure S5A present the overall COG functional classification of intestinal microorganisms in the fermentation solution after sample fermentation. A total of 24 related metabolic pathways are depicted, including translation, ribosomal structure, and biogenesis, transcription, secondary metabolite biosynthesis, transport, and catabolism, replication, recombination, and repair, among others. These pathways provide insights into the metabolic activities of the microbial community during fermentation.

In predicting metabolic pathway functions, KEGG provides extensive data on metabolic pathways, illustrating how various substances in an organism are transformed and utilized. These metabolic pathway data provide information to predict the potential roles and functions of unknown genes or proteins in the metabolic process [43]. As shown in Figure 8B and Figure S5B, the main metabolic pathways are amino acid metabolism, carbohydrate metabolism, energy metabolism, the endocrine system, the metabolism of cofactors and vitamins, and membrane transport. The global and overview maps constitute the largest proportion, indicating the vigorous metabolic activities of the intestinal microbial microbiota and their extensive involvement in metabolic pathways. This promotes the provision of more nutrients to the host, helping to maintain a stable environment in the gut. Furthermore, it may also contribute to the host’s immune function and energy balance, ultimately having a beneficial effect on overall health.

## 4. Conclusions

In this study, we investigated the effects of pH on the gelling and digestive properties of FG–κC and FG–XG, as well as its impact on intestinal microbiota. The results indicated that both the FG–κC and FG–XG groups exhibited a higher gel strength, hardness, gumminess, chewiness, and viscosity compared to FG within the pH range of 4.0–9.0. Specifically, FG–κC6 and FG–XG5 exhibited the highest gelling properties and viscosity. Structural analysis revealed that pH modulated the gel network of FG by altering the hydrogen bonds and protein conformation. There was a positive correlation between gelling properties and the density of the gel network; higher gelling properties corresponded to denser gel networks with a smaller pore size. Compared to FG–κC, the FG–XG complex exhibited superior in vitro digestion properties. Furthermore, both FG–κC6 and FG–XG5 significantly increased the abundance of beneficial bacteria, as evidenced by the elevated densities of the phyla *Proteobacteria* and *Bacteroidetes*, as well as the genus levels of *Lachnospiraceae* and *Bactcroides,* indicating high application value. In conclusion, depending on the desired outcome, whether it is enhancing gel properties, improving viscosity, or optimizing beneficial bacteria, the choice between κC and XG can be tailored accordingly. However, further research is necessary to explore the impact of prepared FG–AP on real food products, such as yoghurt and surimi gels, and to assess its impact on the sensory quality of the final products. Additionally, it is crucial to address issues such as how changes in the gut microbiota can be influenced.

## Figures and Tables

**Table 1:** The pH influence of gel strength and textural characteristics of FG–AP complex gels.

Sample	Gel Strength/g	Hardness/N	Gumminess/N	Chewiness/N	Resilience	Springiness	Cohesiveness
**FG**	244.21 ± 57.53 ^g^	3.55 ± 0.78 ^g^	3.29 ± 0.71 ^e^	2.27 ± 1.14 ^e^	0.74 ± 0.37 ^de^	0.69 ± 0.19 ^b^	0.90 ± 0.05 ^bc^
**FG–κC4**	430.56 ± 15.05 ^f^	7.26 ± 0.06 ^ef^	6.79 ± 0.12 ^d^	6.41 ± 1.70 ^cd^	0.75 ± 0.07 ^cd^	0.97 ± 0.07 ^a^	0.91 ± 0.15 ^ab^
**FG–κC5**	450.67 ± 19.77 ^def^	8.12 ± 0.59 ^de^	7.62 ± 0.52 ^cd^	7.00 ± 0.53 ^bcd^	0.80 ± 0.27 ^ab^	0.94 ± 0.08 ^a^	0.91 ± 0.02 ^a^
**FG–κC6**	537.14 ± 23.16 ^a^	10.46 ± 0.48 ^a^	9.80 ± 0.51 ^a^	9.12 ± 0.54 ^a^	0.78 ± 0.15 ^bc^	0.96 ± 0.12 ^a^	0.91 ± 0.06 ^a^
**FG–κC7**	498.37 ± 6.99 ^abc^	8.76 ± 0.83 ^cd^	8.12 ± 0.77 ^bc^	7.49 ± 0.56 ^bc^	0.80 ± 0.13 ^ab^	0.95 ± 0.52 ^a^	0.91 ± 0.06 ^a^
**FG–κC8**	500.70 ± 38.07 ^abc^	9.56 ± 0.39 ^abc^	8.99 ± 0.40 ^ab^	8.26 ± 0.37 ^ab^	0.79 ± 0.07 ^ab^	0.95 ± 0.24 ^a^	0.92 ± 0.09 ^a^
**FG–κC9**	509.86 ± 44.44 ^abc^	9.66 ± 0.42 ^abc^	9.17 ± 0.38 ^ab^	8.49 ± 0.61 ^ab^	0.82 ± 0.13 ^a^	0.95 ± 0.28 ^a^	0.92 ± 0.05 ^a^
**FG**	244.21 ± 57.53 ^g^	3.55 ± 0.78 ^g^	3.29 ± 0.71 ^e^	2.27 ± 0.11 ^e^	0.74 ± 0.37 ^de^	0.69 ± 0.19 ^b^	0.90 ± 0.05 ^bc^
**FG–XG4**	482.76 ± 28.02 ^cde^	9.11 ± 0.49 ^bcd^	8.34 ± 0.27 ^cd^	7.37 ± 0.48 ^bc^	0.66 ± 0.05 ^g^	0.93 ± 0.33 ^a^	0.88 ± 0.19 ^d^
**FG–XG5**	532.77 ± 20.69 ^ab^	9.90 ± 0.23 ^a^	9.16 ± 0.07 ^ab^	8.43 ± 0.08 ^ab^	0.71 ± 0.08 ^f^	0.95 ± 0.10 ^a^	0.89 ± 0.20 ^c^
**FG–XG6**	490.69 ± 4.43 ^bcd^	9.48 ± 0.54 ^ab^	8.85 ± 0.46 ^bc^	8.35 ± 0.36 ^ab^	0.72 ± 0.13 ^ef^	0.97 ± 0.10 ^a^	0.91 ± 0.03 ^abc^
**FG–XG7**	466.37 ± 8.74 ^cdef^	8.73 ± 0.35 ^cd^	8.22 ± 0.34 ^cd^	7.73 ± 0.38 ^abc^	0.78 ± 0.04 ^bc^	0.97 ± 0.35 ^a^	0.92 ± 0.09 ^a^
**FG–XG8**	442.91 ± 14.28 ^ef^	8.85 ± 0.08 ^d^	8.47 ± 0.25 ^c^	7.63 ± 2.12 ^d^	0.81 ± 0.07 ^a^	0.87 ± 0.14 ^a^	0.92 ± 0.04 ^a^
**FG–XG9**	487.57 ± 10.03 ^cd^	9.18 ± 0.13 ^bc^	9.14 ± 0.16 ^a^	9.09 ± 0.85 ^a^	0.81 ± 0.11 ^a^	0.97 ± 0.14 ^a^	0.93 ± 0.09 ^a^

**Note:** FG–κC4, FG–κC5, FG–κC6, FG–κC7, FG–κC8, and FG–κC9: the pH of FG–κC solution was 4.0, 5.0, 6.0, 7.0, 8.0, and 9.0, respectively. FG–XG4, FG–XG5, FG–XG6, FG–XG7, FG–XG8, and FG–XG9: the pH of FG–XG solution was 4.0, 5.0, 6.0, 7.0, 8.0, and 9.0, respectively. The different lowercase letters in the same column indicated significant differences within the group; Different lowercase letters indicated significant difference between groups (*p* < 0.05).

## Data Availability

The original contributions presented in the study are included in the article/Supplementary Material, further inquiries can be directed to the corresponding authors.

## Supplementary Materials

The following supporting information can be downloaded at: https://www.mdpi.com/article/10.3390/foods14152631/s1, Figure S1: Micro-structured pore size distribution of FG, FG−κC, and FG−XG.; Figure S2: Microbial alpha diversity in fermentation broth after 24 h fermentation. Figure S3: Principal component analysis and hierarchical cluster analysis of Initial group, Control, FG, FG−κC6, and FG−XG5 groups. Figure S4: Heatmap of gut microbial composition at phylum level (A) and genes level (B) in the fermentation fluids of different samples after fermentation. Figure S5: Heatmap of functional predictions for microbial genes in fermentation liquids. (A) Heatmap of COG functional classifications of intestinal microbiota in fermentation fluids. (B) Heatmap of KEGG functional classifications of intestinal microbiota in fermentation fluids. Table S1: Power-law model fitting analysis and η50 values of FG-AP complex at different pH; Table S2: The results of frequency sweep fitting Power-low; Table S3: The composition (%) of β-sheet (Low Frequency), Random structure and α-helix, β-turn and β-sheet (High Frequency) in secondary structure of FG, FG−XG5 and FG−κC6.
