# Supplementary material for: Influences of pH on Gelling and Digestion–Fermentation Properties of Fish Gelatin–Polysaccharide Hydrogels
Source: Foods. 2025 Jul 26;14(15):2631. doi: 10.3390/foods14152631 (PMC12346719; doi:10.3390/foods14152631)
Supplement: Supplementary file 1 [file foods-14-02631-s001.zip › foods-3729895-supplementary.pdf]

Supplementary Information

Influences of pH on gelling and digestion-fermentation properties of fish gelatin-polysaccharide hydrogels

Wanyi Sun [a], Jiajing Chen [a], Xinxin Fan [a], Qiuyu Lu [a], Shengnan Zhan [a,b] , Wenge Yang [a], Tao Huang [a,b]* and Fulai Li [b]*

[a] Zhejiang–Malaysia Joint Research Laboratory for Agricultural Product Processing and Nutrition, College of Food Science and Engineering, Ningbo University, Ningbo, Zhejiang Province, 315211, China
[b] Institute of Drug Discovery Technology, Ningbo University, Zhejiang Province, 315211, China

* Correspondence: author: Tao Huang, cdhuangtao@163.com; Fulai Li, lifulai@nbu.edu.cn

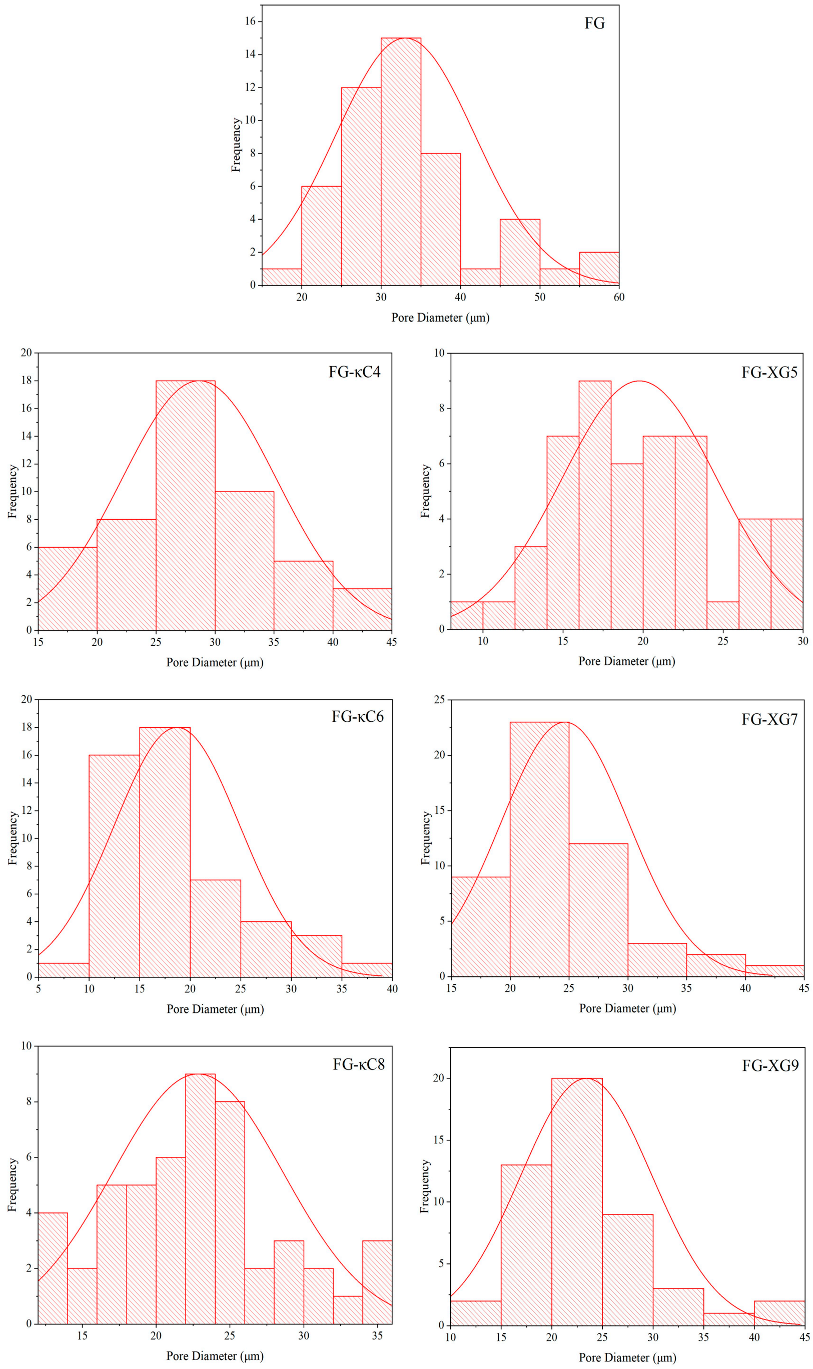

Figure S1. Micro-structured pore size distribution of FG, FG-κC, and FG-XG.

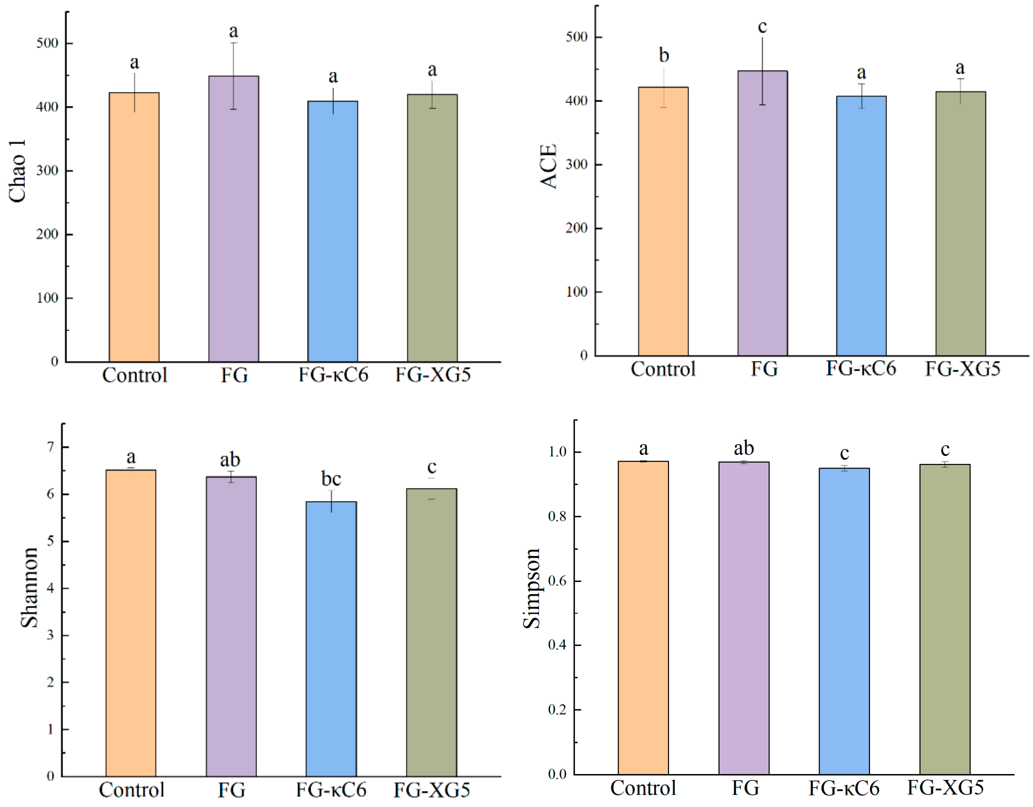

Figure S2 Microbial alpha diversity in fermentation broth after 24 h fermentation

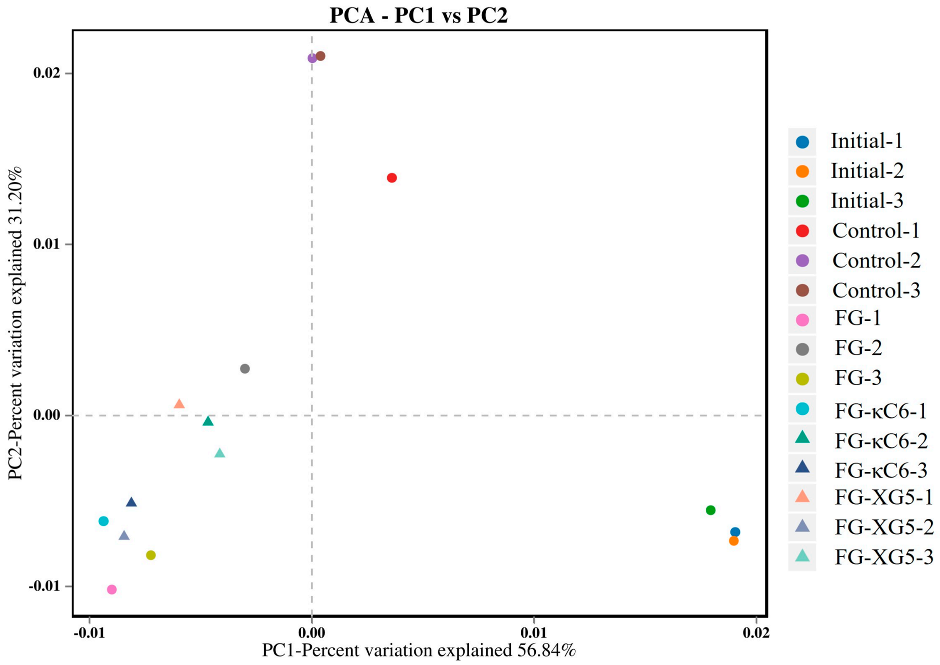

Figure S3. Principal component analysis and hierarchical cluster analysis of Initial group, Control, FG, FG-κC6, and FG-XG5 groups.

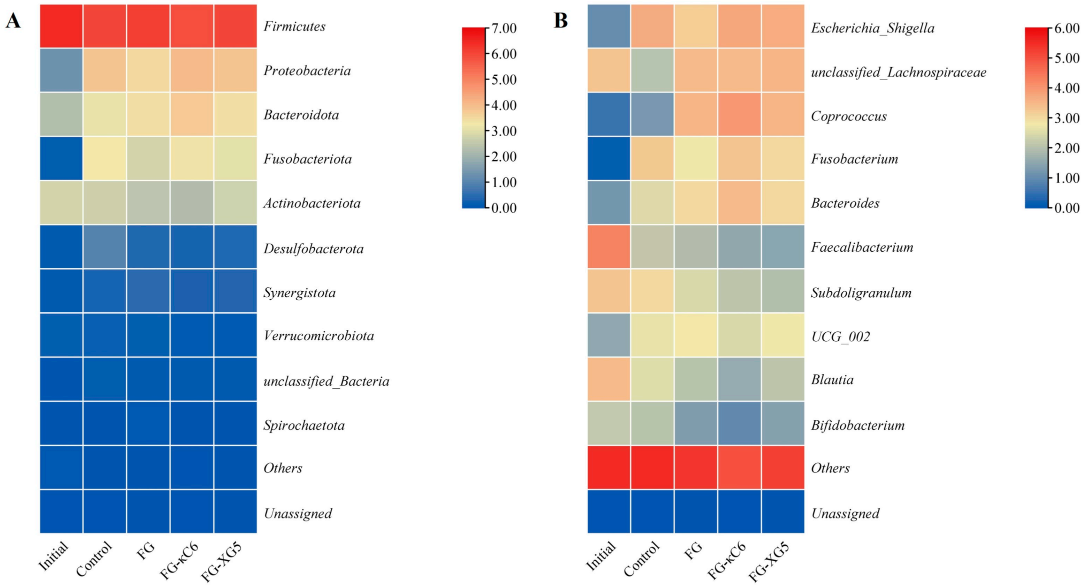

Figure S4. Heatmap of gut microbial composition at phylum level (A) and genes

level (B) in the fermentation fluids of different samples after fermentation.

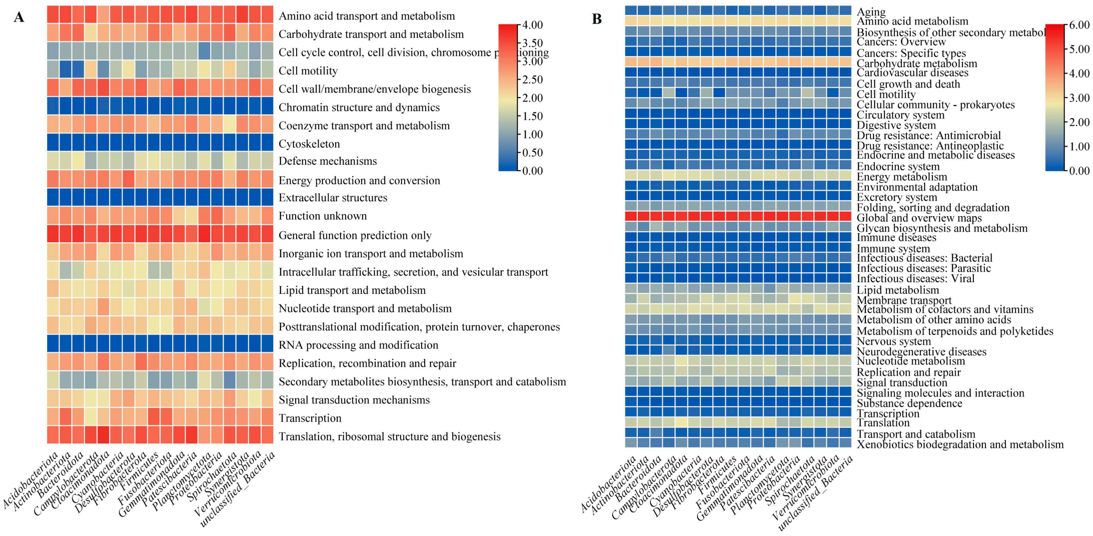

Figure S5. Heatmap of functional predictions for microbial genes in fermentation liquids. (A) Heatmap of COG functional classifications of intestinal microbiota in fermentation fluids. (B) Heatmap of KEGG functional classifications of intestinal microbiota in fermentation fluids.

Table S1 Power-law model fitting analysis and η_{50} values of FG-AP complex at different pH

Samples	η_{50} (Pa·s)	Power-law			
		k	n	R^2	RMSE
**FG**	9.910×10^{-3}	0.00937	0.99806	0.99992	0.018379
**FG-κC4**	1.019×10^{-2}	0.00945	0.98805	0.99957	0.040333
**FG-κC5**	5.502×10^{-2}	0.13105	0.7704	0.99991	0.060182
**FG-κC6**	1.034×10^{-1}	0.46131	0.58995	0.99992	0.061384
**FG-κC7**	8.146×10^{-2}	0.23856	0.71698	0.9998	0.115841
**FG-κC8**	3.254×10^{-2}	0.05535	0.86496	0.99996	0.033616
**FG-κC9**	6.973×10^{-2}	0.22359	0.68272	0.99997	0.033458
**FG-XG4**	1.072×10^{-2}	0.94596	0.42672	0.95983	0.979709
**FG-XG5**	1.246×10^{-2}	0.63316	0.5432	0.99619	0.41804176
**FG-XG6**	1.029×10^{-2}	0.48502	0.5659	0.9975	0.301806
**FG-XG7**	1.052×10^{-2}	0.44954	0.59352	0.99859	0.25305
**FG-XG8**	1.043×10^{-2}	0.25795	0.65237	0.99857	0.215436
**FG-XG9**	1.147×10^{-2}	0.40952	0.64693	0.99925	0.23978

Table S2 The results of frequency sweep fitting Power-low

Sample	G_0	N	R^2
**FG**	4332.01829 ± 8.12331	0.00938 ± 7.89575E-4	0.91016
**FG-κC4**	3373.97098 ± 4.68122	0.0107 ± 5.85587E-4	0.95992
**FG-κC6**	2744.88126 ± 6.35554	0.0132 ± 9.81606E-4	0.92849
**FG-κC8**	1882.11291 ± 1.80141	0.0156 ± 4.07513E-4	0.99057
**FG-XG5**	4267.0145 ± 6.83951	0.01072 ± 6.76534E-4	0.94732
**FG-XG7**	3449.86069 ± 6.21946	0.01308 ± 7.64131E-4	0.9546
**FG-XG9**	3660.4883 ± 6.87451	0.01075 ± 7.92711E-4	0.92957

Table S3. The composition (%) of β-sheet (Low Frequency), Random structure and α-helix, β-turn and β-sheet (High Frequency) in secondary structure of FG, FG-XG5 and FG-κC6.

	β-sheet (Low Frequency, 1640-1600 cm^{-1})	Random structure and α-helix (1600-1640 cm^{-1})	β-turn (1690-1660 cm^{-1})	β-sheet (High Frequency, 1700-1690 cm^{-1})
FG	26.7	30.1	38.1	5.1
FG-XG5	17.78	15.50	51.57	15.15
FG-κC6	14.8	4.50	42.7	38.3
